# Increasing Adoption and Utility of Smartwatches in Older Adults

**DOI:** 10.1093/geroni/igaa057.1326

**Published:** 2020-12-16

**Authors:** Mitchell Roberts, Erica Sappington, Ali Yalcin, Jeffrey Lowenkron, David Amzallag, Carla VandeWeerd

**Affiliations:** 1 University of South Florida, Tampa, Florida, United States; 2 The Villages Health, The Villages, Florida, United States; 3 Aviv Scientific, Bnei Brak, Israel

## Abstract

Older adults (55+) represent one of the fastest growing groups adopting wearable devices (e.g. smartwatches). However, questions remain as to how older adults, healthcare providers and researchers can maximize the ability of these devices to maintain and improve health. The objective of this study was to understand the needs and preferences of older adults in wearable devices, as part of a Florida High Tech Corridor Matching Grant between The Villages Health, Aviv/Xtend Scientific, and The University of South Florida. Six focus groups were conducted with older adults (n=36) living in The Villages, Florida, and were stratified by gender. Topics such as adoption, benefits/concerns, usability, and potential gender differences were explored. Heart rate monitoring, calories burned, and step counting features were most often utilized by current wearable users (43%). Participants reported that the most important benefits of wearables were atrial fibrillation identification, fitness tracking and fall detection. Concerns included privacy, the “learning curve” and too frequent of notifications by the device. Across both genders, choice resonated strongly as a theme. Male and female participants desired personalized, easy to understand outcomes and control over data sharing preferences. Men were interested in continual access to wearable health data, whereas females were interested in daily or weekly summaries. Further, men reported higher on-going use and comfort with smartwatches as compared to females. Participants suggested “low, medium, and high-tech savvy” user profiles and supporting resources (e.g. 1-1 training, paper manuals) to enhance adoption. Recommendations for policy and practice are shared, in light of the findings.

